# Signatures of oral microbiome in HIV-infected individuals with oral Kaposi's sarcoma and cell-associated KSHV DNA

**DOI:** 10.1371/journal.ppat.1008114

**Published:** 2020-01-17

**Authors:** Marion Gruffaz, Tinghe Zhang, Vickie Marshall, Priscila Gonçalves, Ramya Ramaswami, Nazzarena Labo, Denise Whitby, Thomas S. Uldrick, Robert Yarchoan, Yufei Huang, Shou-Jiang Gao

**Affiliations:** 1 Department of Molecular Microbiology and Immunology, Keck School of Medicine, University of Southern California, Los Angeles, United States of America; 2 Department of Electrical and Computer Engineering, University of Texas at San Antonio, San Antonio, Texas, United States of America; 3 Viral Oncology Section, AIDS and Cancer Virus Program, Leidos Biomedical Research Inc., Frederick National Laboratory for Cancer Research, Frederick, Maryland, United States of America; 4 HIV and AIDS Malignancy Branch, Center for Cancer Research, National Cancer Institute, Bethesda, Maryland, United States of America; 5 Fred Hutchinson Cancer Research Center, Seattle, Washington, United States of America; 6 Department of Epidemiology and Biostatistics, The University of Texas Health San Antonio, San Antonio, Texas, United States of America; 7 UPMC Hillman Cancer Center, Department of Microbiology and Molecular Genetics, University of Pittsburgh, Pittsburgh, Pennsylvania, United States of America; University of Pennsylvania, UNITED STATES

## Abstract

Infection by Kaposi’s sarcoma-associated herpesvirus (KSHV) is necessary for the development of Kaposi’s sarcoma (KS), which most often develops in HIV-infected individuals. KS frequently has oral manifestations and KSHV DNA can be detected in oral cells. Numerous types of cancer are associated with the alteration of microbiome including bacteria and virus. We hypothesize that oral bacterial microbiota affects or is affected by oral KS and the presence of oral cell-associated KSHV DNA. In this study, oral and blood specimens were collected from a cohort of HIV/KSHV-coinfected individuals all previously diagnosed with KS, and were classified as having oral KS with any oral cell-associated KSHV DNA status (O-KS, n = 9), no oral KS but with oral cell-associated KSHV DNA (O-KSHV, n = 10), or with neither oral KS nor oral cell-associated KSHV DNA (No KSHV, n = 10). We sequenced the hypervariable V1-V2 region of the 16S rRNA gene present in oral cell-associated DNA by next generation sequencing. The diversity, richness, relative abundance of operational taxonomic units (OTUs) and taxonomic composition of oral microbiota were analyzed and compared across the 3 studied groups. We found impoverishment of oral microbial diversity and enrichment of specific microbiota in O-KS individuals compared to O-KSHV or No KSHV individuals. These results suggest that HIV/KSHV coinfection and oral microbiota might impact one another and influence the development of oral KS.

## Author summary

Kaposi’s sarcoma (KS) is the most common cancer occurring in HIV-infected individuals worldwide, and often involves the mouth. While infection by Kaposi’s sarcoma-associated herpesvirus (KSHV) is necessary for the development of KS, other cofactors remain unclear. In this study, we evaluated the impact of oral bacterial microbiota on the development of oral KS and the presence of oral cell-associated KSHV DNA by studying a cohort of HIV/KSHV-coinfected individuals all previously diagnosed with KS, classified as having oral KS with any oral cell-associated KSHV DNA status (O-KS), no oral KS but with oral cell-associated KSHV DNA (O-KSHV), or with neither oral KS nor oral cell-associated KSHV DNA (No KSHV). We observed impoverishment of oral microbial diversity and enrichment of specific types of microbes in O-KS individuals compared to O-KSHV or No KSHV individuals. Hence, HIV/KSHV coinfection and oral microbiota might impact one another and influence the development of oral KS.

## Introduction

Infection by Kaposi’s sarcoma-associated virus (KSHV), also called human herpesvirus-8 (HHV-8), is associated with several human malignancies or hyperinflammatory conditions including Kaposi’s sarcoma (KS), primary effusion lymphoma (PEL), multicentric Castleman’s disease (MCD) and KSHV inflammatory cytokine syndrome (KICS)[1, 2]. KSHV is a gammaherpesvirus having latent and lytic replication phases[3]. KSHV DNA can often be detected in oral cells of individuals with asymptomatic KSHV infection[4]. In KSHV-associated malignancies, KSHV latent infection and latent genes are essential for the proliferation, survival and immune evasion of tumor cells[1, 5]. However, KSHV lytic replication, detected in a small proportion of cells within tumors, also contributes to disease pathogenesis[1, 5].

KS is the most common KSHV-associated malignancy worldwide. It is characterized by the proliferation of vascular spindle tumors cells, and extensive inflammatory infiltration and angiogenesis[1]. AIDS-associated KS (AIDS-KS) may affect the skin, the oral cavity, lymph nodes and internal organs including gut, stomach, liver and lung. Oral KS is the first manifestation in 20% of AIDS-KS individuals[6]. Before the introduction of effective antiretroviral therapy, up to 70% of AIDS-KS individuals in the US eventually developed oral, visceral or cutaneous KS[6]. Antiretroviral therapy effectively decreases KS incidence[7] and the presence of KSHV in oral cells[8]. However, even in the era of combination antiretroviral therapy (cART), KS remains one of the most common AIDS-related cancers in the United States and throughout sub-Saharan Africa[9]. Upon initiation of cART, KS can also progress, possibly triggered by, or exacerbated by a KS immune reconstitution inflammatory syndrome (IRIS)[10]. AIDS-KS continues to have a high mortality rate in sub-Saharan Africa[11] and is still associated with morbidity and mortality in the US among HIV-infected patients[12].

The development of next generation sequencing (NGS) has enabled the identification and quantification of the microbiome including bacteria, viruses and fungi in healthy individuals, or in a context of disease[13]. Indeed, numerous metagenomics studies of the microbiome have highlighted microbial pattern modifications in various types of cancer and viral infections. Particularly, over the course of HIV infection, microbial diversity is altered as a result of host-microbiota interactions[14]. Moreover, in cancer, specific microbial DNA signatures have been identified[15]. For example, two bacteria commonly associated with gastric and colorectal cancers in humans are *Helicobacter pylori* and *Fusobacterium nucleatum*, respectively[16, 17]. Other studies also showed a positive association between *Propionibacterium acnes* and the development of prostate cancer[18]. Some enterotoxic *Escherichia coli* (*E*. *coli*) strains are associated with tumorigenesis in mouse models of colorectal cancers[19]. We have recently shown that *E*. *coli* C25 strain could promote KSHV-induced tumorigenesis in a KS-like mouse model[20].

Numerous pathogenic mechanisms, including the production of genotoxic inducers, the activation of TLRs and pro-inflammatory pathways, the inhibition of the immune response, and the increase of the cellular turnover have been proposed to explain the oncogenic properties of some types of bacteria[15]. Bacterial byproducts such as short-chain fatty acids (SCFA) that are highly abundant in individuals suffering from periodontal disease can induce KSHV reactivation[21, 22]. Interestingly, HIV-infected individuals display a higher rate of periodontal disease[23], which has been proposed to promote oral KS development by inducing pro-inflammatory cytokines or releasing SCFA. We previously showed that *E*. *coli* infection or bacterial ligands such as LPS can promote KSHV-induced tumorigenesis in mice through the activation of TLR4 and pro-inflammatory pathways[20].

In this study, we examined the oral bacterial microbiota in a cohort consisting of HIV/KSHV-coinfected individuals all previously diagnosed with KS, with or without oral KS whose status of oral cell-associated KSHV DNA was assessed. We showed a diminution of the oral microbial diversity and enrichment of specific bacteria in HIV/KSHV-coinfected individuals with oral KS, which was independent of the presence of oral cell-associated KSHV DNA. Hence, the composition of the oral microbiota may contribute to the development of oral KS.

## Results

### Cohort characteristics and clinical status

We recruited 29 HIV/KSHV-coinfected individuals with a history of pathology-confirmed KS by physician physical examination, including 9 individuals who had oral KS involvement with any oral cell-associated KSHV DNA status (O-KS), 10 individuals who had detectable intermittent oral cell-associated KSHV DNA without oral KS (O-KSHV), and 10 individuals who had neither oral cell-associated KSHV DNA nor oral KS (No KSHV) (**Table 1**). While all individuals had KS when admitted into the cohort, 5 of these subjects no longer had detectable KS at the time of sampling but had either KSHV-MCD or PEL (**Table 1**). Intermittent oral cell-associated KSHV DNA was defined based on the detection of KSHV DNA in oral cells in longitudinal follow-ups in at least 3 visits. Oral microbiota, oral cell-associated KSHV DNA, KSHV blood DNA detected in peripheral blood mononuclear cells (PBMC), and HIV load were examined at the time of sampling while CD4+ T cell count and CD8+ T cell count were obtained from medical records. Of note, while all individuals were infected with HIV when they were admitted into the cohort, only nine had detectable (>50 copies/mL) HIV RNA at the time of sampling, two of whom had >400 copies/ml. Two individuals in the O-KSHV group were negative for oral cell-associated KSHV DNA at the time of sampling; however, they had a significant history of detectable oral cell-associated KSHV DNA with one individual positive for 17 of 20 time points (85%) tested over a 43 months period and the second individual positive for 8 of 15 time points (53%) tested over a 55 months period. All individuals were men and had a median age of 45 years.

**Table 1:** Clinical characteristics of HIV/KSHV-coinfected individuals.

	Sub-ject	KS status at the time of sampling	HIV antiretroviral therapy (ART)	HIV ART duration (month)	Time from HIV diagnosis (month)	CD4 (cells/μl)	CD8 (cells/μl)	HIV in blood (copies/ml)	PBMC KSHV DNA (copies/10^6^ cells)	Oral cell-associated KSHV DNA (copies/10^6^ cells)
**GROUP 1:** **O-KS** Oral KS	1	O-KS	Darunavir, Tenofovir, Emtricitabin, Ritonavir	3.2	3.9	46	475	1150	QP	<3
	2	O-KS	Tenofovir, Emtricitabin, Efavirenz	8.4	8.4	47	1099	<50	QP	<3
	3	O-KS	Tenofovir, Emtricitabine, Ritonavir, Atazanavir	15.9	24.1	106	423	74	ND	58650
	4	O-KS	Abacavir, Etravirine, Raltegravir	6.4	7.3	49	113	<20	QP	25
	5	O-KS	Elvitegravir, Cobicistat, Emtricitabine, Tenofovir	110.6	110.6	753	424	<20	QP	1365000
	6	O-KS	Abacavir, Dolutegravir, Lamivudine	260.0	261.0	39	402	<20	QP	<3
	7	O-KS	Abacavir, Dolutegravir, Lamivudine	6.8	9.4	151	1890	<20	<3	<3
	8	O-KS	Emtricitabine, Tenfovir, Rilpivirine	0	127.7	6	389	156292	122600	5650
	9	O-KS	Abacavir, Lamivudine, Dolutegravir	2.4	30.8	16	500	<20	3400	32900
**GROUP 2: O-KSHV** No oral KS, intermittent detection of oral cell-associated KSHV DNA	10	No KS	Efavirenz, Emtricitabine,Tenofovir	219.2	295.2	571	1288	<20	24500	1640
	11	KS	Atazanavir, Lamivudine, Zidovudine	45.8	91.9	637	845	<50	<3	157000
	12	KS	Lamivudine, Zidovudine, Lopinavir, Ritonavir	33.3	40.8	119	937	<20	QP	185
	13	KS	Efavirenz, Tenofovir, Emtricitabine	6.0	131.8	285	463	<50	<3	38000
	14	KS	Tenofovir, Emtricitabine, Dolutegravir	241.2	241.2	180	382	<20	350	<3
	15	KS	Abacavir, Dolutegravir, Lamivudine	187.6	189.6	449	555	<20	<3	36000
	16	KS	Ritonavir, Tenofovir, Emtricitabine, Darunavir	5.2	26.2	307	1112	75	2000	4400
	17	No KS	Tenofovir, Emtricitabine, Ritonavir, Reyataz	187.5	190.5	584	1148	<50	570	<3
	18	No KS	Raltegravir, Darunavir, Ritonavir, Tenofovir, Emtricitabine	279.1	315.2	594	1700	<20	42000	1300
	19	KS	Efavirenz, Emtricitabine, Tenofovir	17.7	53.8	287	718	<50	270500	2200
**GROUP 3: No KSHV** No oral KS, no detection of oral cell-associated KSHV DNA*	20	KS	Efavirenz, Emtricitabine, Tenofovir	201.5	225.6	215	479	<20	<3	<3
	21	No KS	Efavirenz, Emtricitabine, Tenofovir	49.0	55.0	386	625	<20	QP	<3
	22	KS	Tenofovir, Emtricitabine, Raltegravir	39.4	147.6	396	667	<50	<3	<3
	23	KS	Ritonavir, Lamivudine, Abacavir, Darunavir	68.3	69.5	465	712	<20	<3	<3
	24	KS	Tenofovir, Emtricitabine, Raltegravir	3.6	67.4	185	842	27	QP	<3
	25	No KS	Tenofovir, Emtricitabine, Efavirenz	29.5	286.5	378	1493	336	<3	<3
	26	KS	Abacavir, Lamivudine, Zidovudine, Atazanavir	58.7	185.8	482	385	<50	<3	<3
	27	KS	Norvir, Tenofovir, Emtricitabine, Darunavir	81.7	81.7	437	1308	58	240	<3
	28	KS	Raltegravir, Darunavir, Etravirine, Ritonavir	189.3	249.4	97	518	109	500	<3
	29	KS	Lamivudine, Tenofovir, Nelfinavir	183.0	183.0	445	950	447	60	<3

ND: Not done.

QP: Qualitative positive, i.e. detectable KSHV DNA <3 copies/10^6^ cells (assay cut-off).

*: No detectable oral cell-associated KSHV DNA over 3 or more time points.

**Fig 1:**
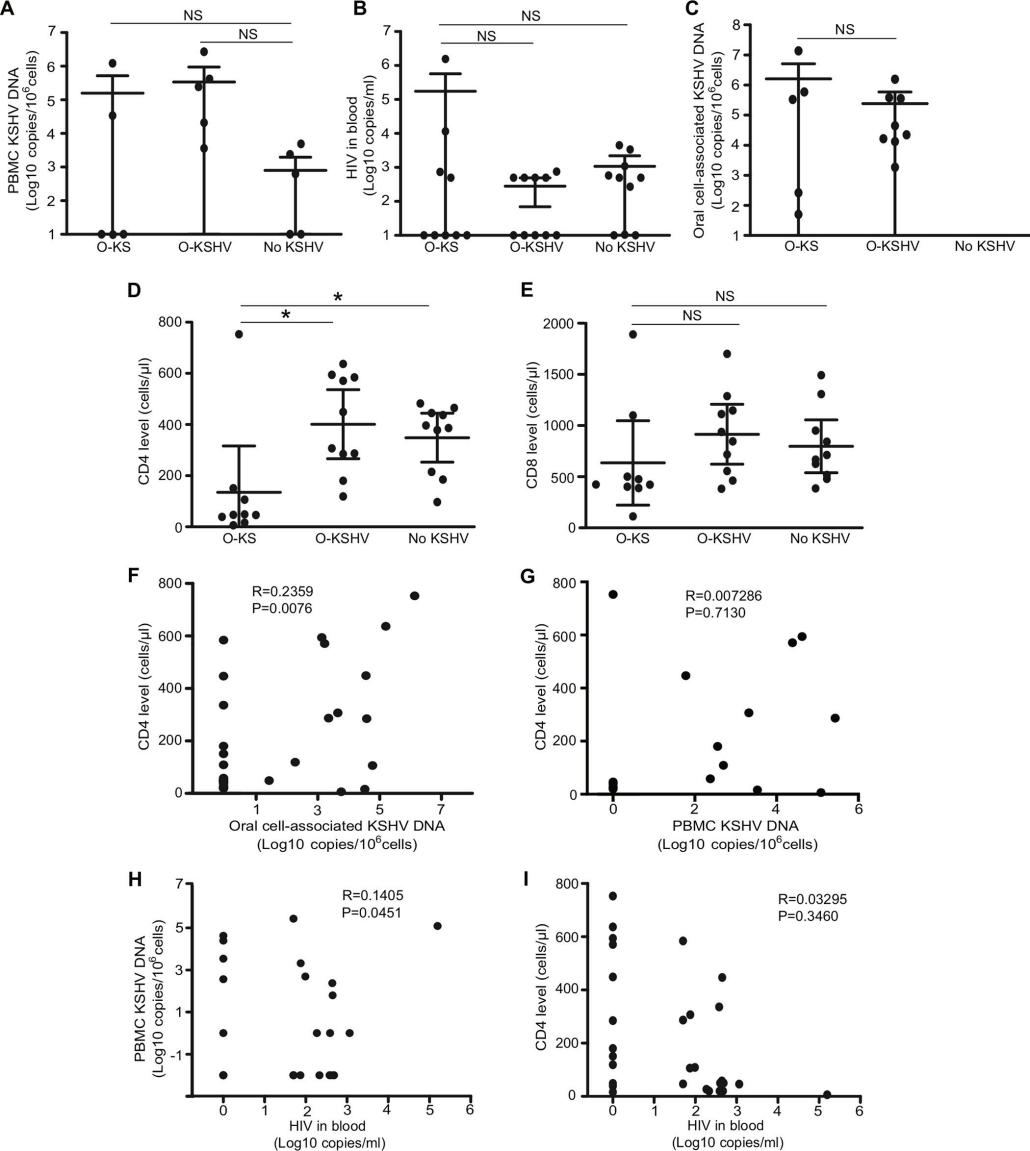
Viral and immunological status in three studied groups of HIV/KSHV-coinfected individuals. (A-C) Quantification of PBMC KSHV DNA (A), HIV load (B), and oral cell-associated KSHV DNA (C). (D-E) Quantification of levels of CD4+ T cell count (D) and CD8+ T cell count (E). (F) Correlation of CD4+ T cell count with oral cell-associated KSHV DNA. (G) Correlation of CD4+ T cell count with PBMC KSHV DNA. (H) Correlation of HIV load and PBMC KSHV DNA. (I) Correlation of CD4+ T cell count with HIV load. P-value ≤0.05 (*) was considered as significant. NS indicates not significant (P-value ≥0.05).

Few individuals had detectable KSHV in PBMC and no significant difference between the studied groups was observed (**Fig 1A**). Likewise, no significant difference in HIV load (**Fig 1B**) was noted between groups. There were 5 of 9 and 8 of 10 individuals who had detectable oral cell-associated KSHV DNA in the O-KS and O-KSHV groups, respectively, but no significant difference was observed between the O-KSHV and O-KS groups at the time of collection (**Fig 1C)**.

The CD4+ T cells was significantly lower in the O-KS group than in the other two groups (**Fig 1D**), and 8 of 9 individuals in the O-KS group had advanced immunodeficiency with a CD4+ T cell count <200 cells/μL. However, no difference of CD8+ T cell count was observed between the O-KS and either of the other two groups (**Fig 1E**). There was a significant positive correlation between oral cell-associated KSHV DNA and CD4+ T cell count (**Fig 1F**) as previously described[8, 24]. However, there was no association between CD4+ T cell count and PBMC KSHV DNA (**Fig 1G**). There was a weak positive correlation between HIV load and PBMC KSHV DNA (**Fig 1H**) as previously described[25]. No correlation between CD4+ T cell count and HIV load was observed (**Fig 1I**).

### Microbiota richness and diversity across samples

**Fig 2:**
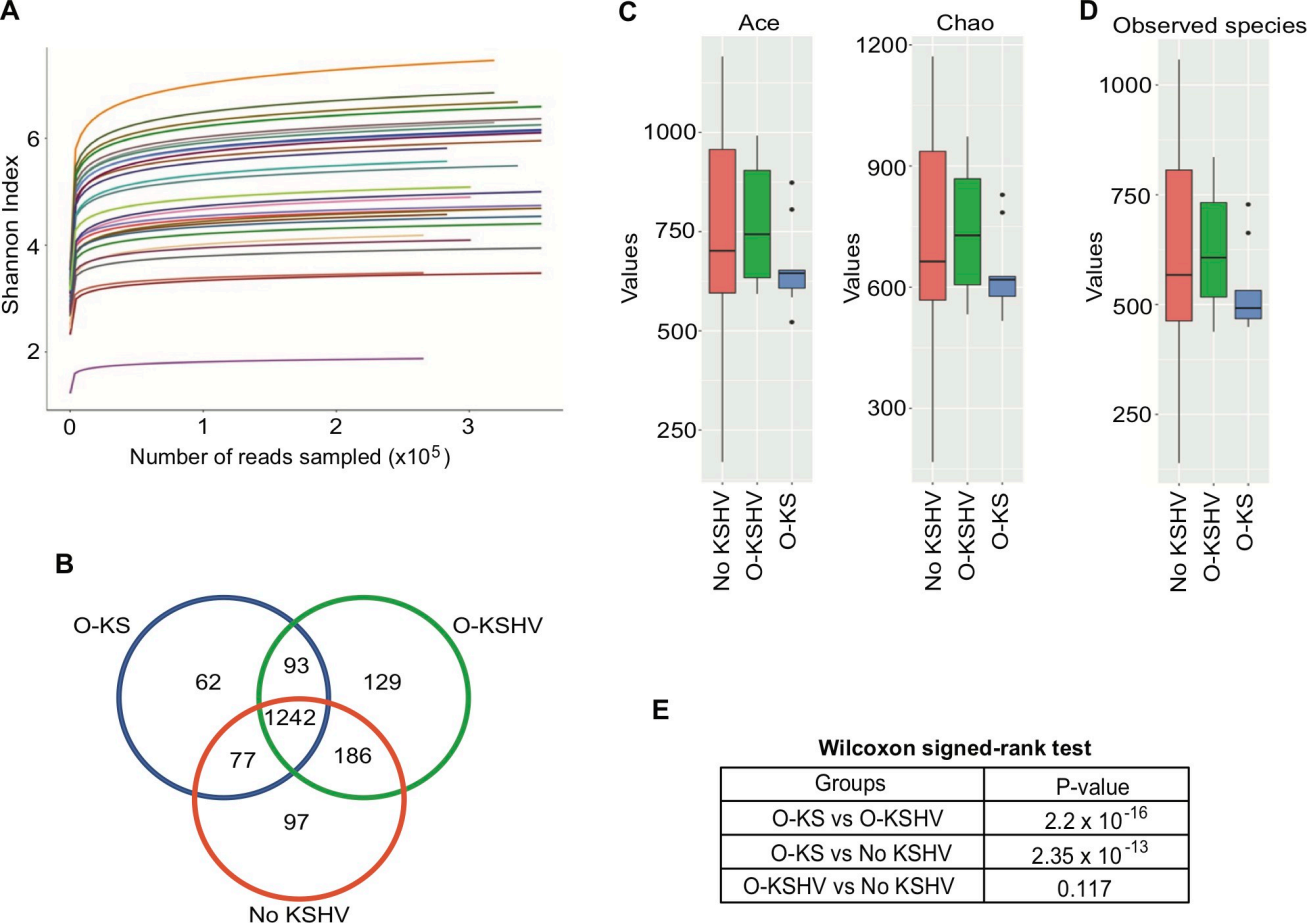
The richness and diversity of microbiota in three studied groups of HIV/KSHV-coinfected individuals. (A) Shannon index measuring how evenly OTUs are distributed in a sample by showing the numbers of reads for all specimens at species level. Each specimen is represented by a color. (B) Venn diagram of OTUs identified in the three studied groups at species level. (C) Diversity across specimens at species level for the three studied groups examined with the Observed Species Algorithm. (D) The richness across different specimens at species level in all studied groups examined with the Ace and Chao Algorithms. (E) Examination of the differences across all three studied groups at species level using Wilcoxon signed-ranked test.

We sequenced the hypervariable V1-V2 region of 16S rRNA gene from oral specimens of the 29 individuals using an Illumina MiSeq system. A total of 15,261,199 raw sequences were generated. After quality control and filtering, 13,098,094 high-quality sequences with an average length of 263 bp were recovered for further analysis, with an average of 451,658 reads per sample (ranging from 333,131 to 569,690 reads). After alignment with QIIME database, unique representative sequences were classified into 1,886 operational taxonomic units (OTUs), from which 16 phyla, 28 classes, 41 orders, 79 families, 125 genera and 148 species were identified. Shannon diversity index was used to evaluate the sequencing depth. All the rarefaction curves reached plateau indicating that there was sufficient sequencing coverage depth (**Fig 2A**). Using Venn diagram, we observed that all 3 groups shared 1,242 OTUs, whereas 62, 129 and 97 OTUs were specific to the O-KS, O-KSHV and No KSHV groups, respectively (**Fig 2B**).

We calculated the species richness (using the Ace and Chao1 nonparametric methods for estimating the number of species in a community) (**Fig 2C**) and the α-diversity of the observed species (using the Shannon index measuring how evenly OTUs are distributed in a sample) among all groups (**Fig 2D**). We observed a strong diminution of the α-diversity and richness in individuals in the O-KS group compared to the other two groups. Using Wilcoxon signed-rank test, we tested the statistical difference between the richness and α-diversity of each pair of groups (**Fig 2E**). We observed a significant dissimilarity between the O-KS and O-KSHV groups (P-value 2.2e-16), and between the O-KS and No KSHV groups (P-value 2.35e-13), whereas the O-KSHV and No KSHV groups were similar (P-value 0.117). Hence, individuals of the O-KS group tend to cluster together and were more distant from individuals of the O-KSHV or No KSHV groups, suggesting an association between the oral microbiota and the presence of oral KS in HIV/KSHV-positive individuals.

### Relative abundances of OTUs and taxonomic compositions of bacterial populations at phylum and species levels across the 3 different groups of HIV/KSHV-coinfected individuals

**Fig 3:**
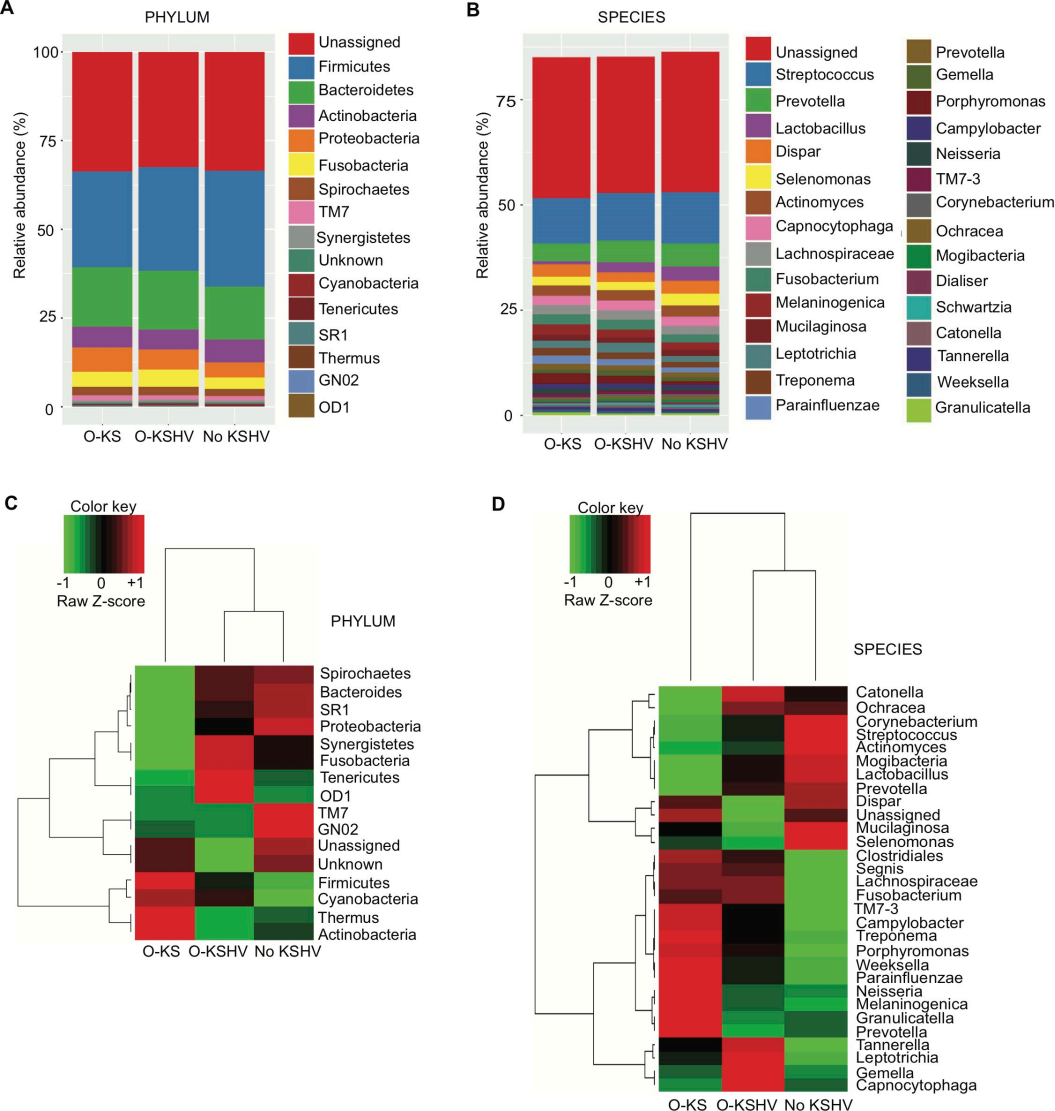
Relative abundances of OTUs and taxonomic compositions of bacterial communities at phylum and species levels in three studied groups of HIV/KSHV-coinfected individuals. (A) Mean of relative OTU abundances at phylum level for the three studied groups. (B) Mean of relative OTU abundances of the 30 most abundant species for the three studied groups. (C) Mean of the relative OTU abundances at phylum level clustered by the three studied groups. (D) Mean of the relative OTU abundances of the 30 most abundant species clustered by the three studied groups.

The relative abundances of OTUs, as well as the taxonomic compositions of bacterial populations in individuals of the 3 studied groups were analyzed at different taxonomic levels. The top 5 most abundant identifiable phyla in the oral microbiota were *Firmicutes*, *Bacteroidetes*, *Actinobacteria*, *Proteobacteria* and *Fusobacteria* (**Fig 3A**), which were consistent with previous reports[26]. At the species level, *Streptococcus*, *Prevotella*, *Lactobacillus*, *Dispar* and *Selenomonas* were the top 5 most abundant species for all 3 groups (**Fig 3B**). To investigate the taxonomic composition of the bacterial populations in all 29 subjects, heatmaps were constructed by clustering each individual OTU compositions (**S1A and S1B Fig**), as well as each mean of relative OTU abundances for the 3 studied groups (**Fig 3C and 3D**). The heatmaps constructed at phylum and species levels demonstrated that the O-KSHV and No KSHV groups clustered together, whereas the O-KS group was phylogenetically more distant from other groups (**Fig 3C and 3D**), indicating that distinct oral microbiome might influence the development of oral KS in HIV/KSHV-coinfected individuals or that the oral microbiome is perturbed by the development of oral KS.

### Alterations of the oral microbiome in HIV/KSHV-coinfected individuals with oral cell-associated KSHV DNA or oral KS

**Fig 4:**
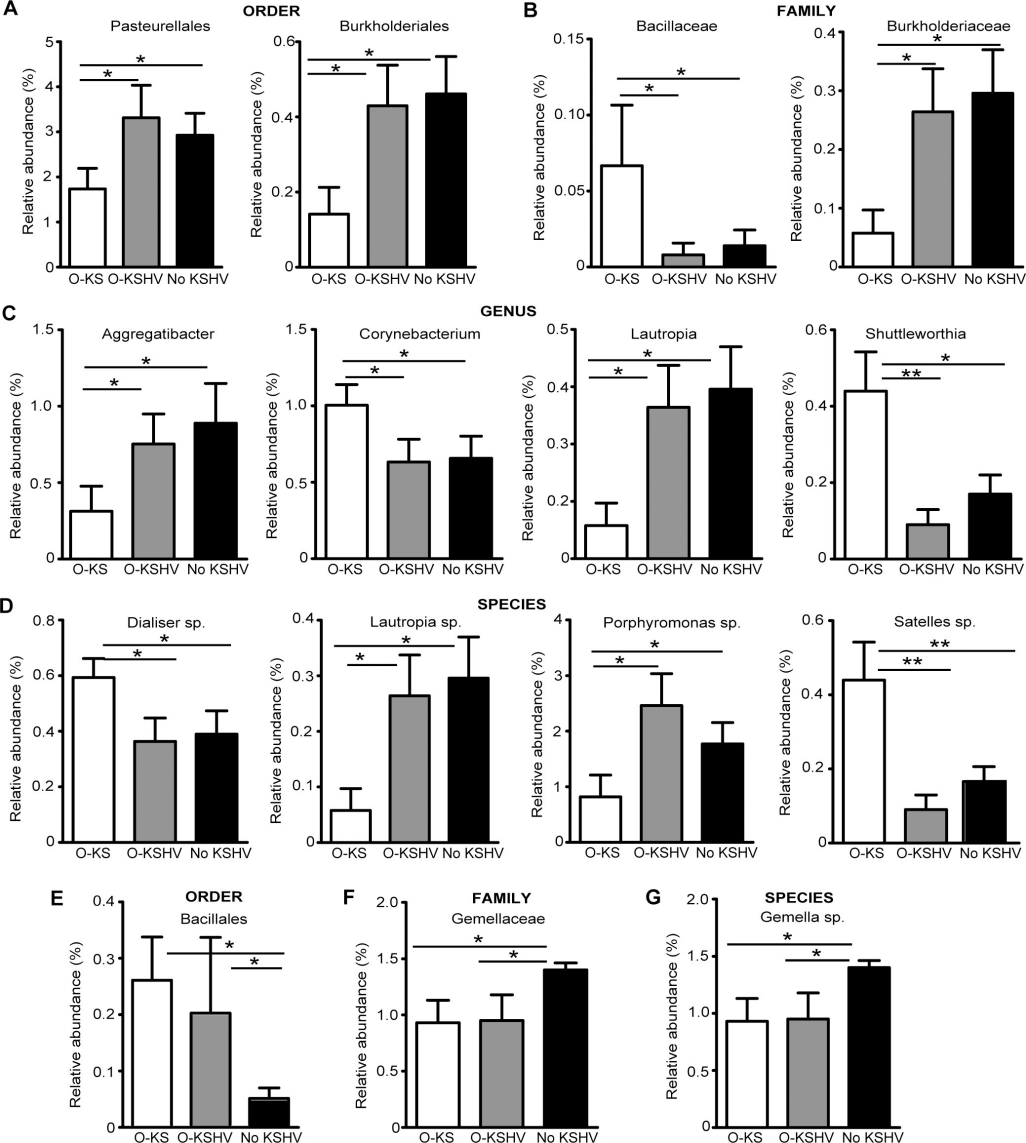
Alterations of oral microbiota in HIV/KSHV-coinfected individuals with oral cell-associated KSHV DNA or oral KS. (A-D) Distinct signatures of O-KS group shown by relative abundances of OTUs at order level (A), family level (B), genus level (C) and species level (D). (E-G) Distinct signatures of No KSHV group shown by relative abundances of OTUs at order level (E), family level (F) and species level (G). Statistical analysis was performed using Student’s t-test with the GraphPad Prism software. Only significant results are represented.

**Fig 5:**
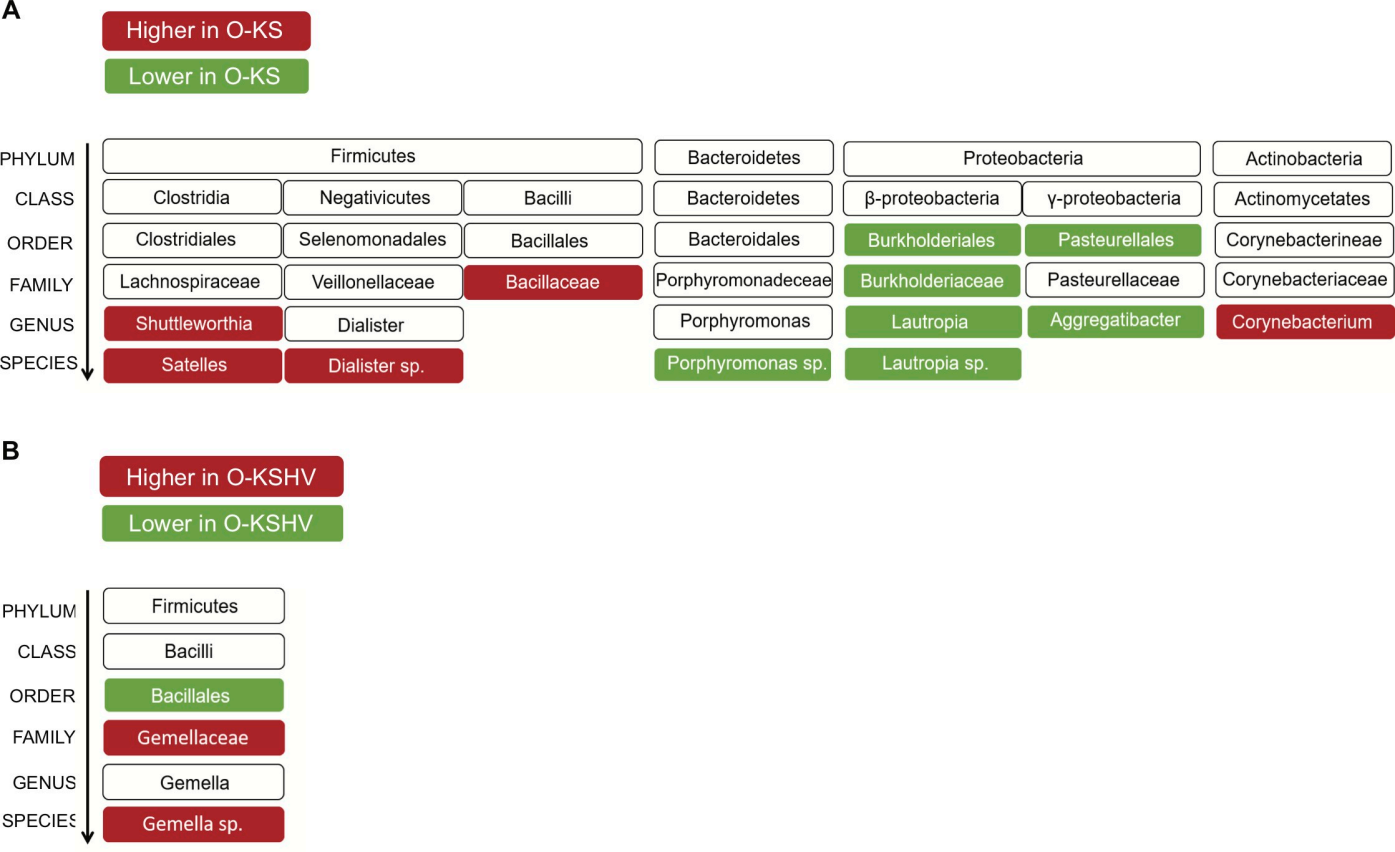
Phylogenetic links among the OTUs altered in HIV/KSHV-coinfected individuals who developed oral KS (A), or with oral cell-associated KSHV DNA (B).

We further examined the differences of bacterial distributions across all 3 studied groups at all phylogenetic levels. We observed a significant diminution of *Pasteurellales* and *Burkholderiales* at order level in the O-KS group compared to the O-KSHV and No KSHV groups (**Fig 4A**). At family level, *Bacillaceae* was enriched in the O-KS group, whereas the abundance of *Burkholderiaceae* decreased (**Fig 4B**). At genus level, the abundances of *Aggregibacter* and *Lautropia* were decreased in the O-KS group, whereas those of *Corynebacterium* and *Shuttleworthia* were increased (**Fig 4C**). At the species level, the abundances of *Dialister* and *Satelles* were increased whereas those of *Lautropia* and *Porphyromonas* were decreased in the O-KS group (**Fig 4D**). No significant differences were observed among all 3 groups at phylum and class levels. The increase in OTUs in the O-KS group clustered in the *Firmicutes* and *Actinobacteria* phylum, whereas the decrease in OTUs in the O-KS group centered in the *Bacteroides* and *Proteobacteria* phylum (**Fig 5A**).

When individual bacterial orders, families, or species groups were examined, there were some significant differences between individuals with oral cell-associated KSHV DNA (O-KSHV) as compared to those without oral cell-associated KSHV DNA (No KSHV). Some of these differences were independent of oral KS status as they were also observed between the O-KS group and the No KSHV group, most likely due to the fact that 5 of 9 individuals in the O-KS group had detectable oral cell-associated KSHV DNA. For examples, the abundance of *Bacillales* order increased in both O-KSHV and O-KS groups (**Fig 4E**), whereas those of *Gemellaceae* family and the *Gemella* species decreased (**Fig 4F and 4G and Fig 5B**).

Altogether, these results indicated that alterations of the oral microbiota might influence the detection of oral cell-associated KSHV DNA and development of oral KS in HIV/KSHV-coinfected individuals, or that the presence of oral cell-associated KSHV DNA and development of oral KS may influence the composition of the oral microbiome.

## Discussion

Altered oral microbiota has been observed in several diseases including diabetes, bacteremia, endocarditis, cancer and autoimmune disease, and in some cases can influence disease progression[27] or tumor response to immunotherapy[28]. Furthermore, we previously demonstrated that TLR4 stimulation with LPS from either *E*. *coli* strain K12 or C25 promoted KSHV-induced cellular transformation and tumorigenesis in a KS-like animal model[20]. Therefore, the purpose of this study was to investigate whether oral KS was associated with an alteration of the bacterial microbiota in the oral cavity. Our results revealed for the first time significant changes in diversity in oral microbiota in HIV/KSHV-coinfected individuals with oral KS compared to those without oral KS. These observations support the close interactions of microbiome, viral infections and cancer development[29, 30], and to our knowledge, it is the first study in the context of a KSHV-associated cancer in HIV-infected individuals.

We observed a strong diminution of microbial α-diversity and richness in individuals developing oral KS compared to the other two groups without oral KS. Imbalance in microbial flora composition is correlated with impaired immune cell activity and the decrease of oral microbial diversity could affect immune responses[31]. We indeed have observed lower CD4+ T cell counts in the O-KS group than the other two groups (**Fig 1D**). The impoverishment in oral microbiota may have implications for the immune reconstitution during cART in HIV-infected patients developing oral KS. In melanoma patients undergoing anti-PD-1 immunotherapy, significant differences were observed in the diversity and composition of the gut microbiome of responders versus non-responders. Higher gut microbiome diversity was associated with improved response to anti-PD-1 immunotherapy in patients with metastatic melanoma[28]. Nevertheless, advanced immunodeficiency can also affect microbial diversity. Further investigations are required to elucidate the role of decreased microbial diversity and alterations of specific microbiota in the development oral KS.

In healthy individuals, the oral microbiota is usually composed of the phyla *Firmicutes*, *Proteobacteria*, *Bacteroidetes*, *Actinobacteria*, and *Fusobacteria*, with a predominance in the genus *Streptococcus* followed by *Prevotella*, *Veillonella*, *Neisseria*, and *Haemophilus*[26]. As observed in the oral cavity of healthy individuals[26], the top 5 phyla in all 3 groups of HIV/KSHV-coinfected individuals consisted of the same 5 phyla (**Fig 3A**). At species level, because of the high variability within each individual, no strict consensus has been identified so far regarding the bacterial relative abundance in the oral cavity in healthy individuals[26, 30]. However, we identified *Streptococcus*, *Prevotella*, *Lactobacillus*, *Dispar sp*. and *Selenomonas* as the top 5 species in all 3 groups of HIV/KSHV-coinfected individuals (**Fig 3B**). Although there was no difference in the distribution of the top 5 phyla or species across all 3 groups (**Fig 3B**), the cluster analysis showed that the phylum and species in the O-KS group were distinct from the O-KSHV and No KSHV groups (**Fig 3C and 3D**), highlighting the modification of the microbial pattern in individuals developing cancer as previously reported[29].

Oral squamous cell cancer (OSCC) is the oral cancer with the highest incidence, and ranked the 15th place in frequency among all types of cancer in 2012[32]. OSCC has been associated with increases of oral *Capnocytophaga gingivalis* and *Prevotella melaninogenica* belonging to the *Bacteroidetes* phylum, and *Streptococcus mitis* belonging to the *Firmicutes* phylum, as well as decreases of *Citrobacter* and *Neisseraceae* belonging to the *Proteobacteria* phylum compared to the cancer-free control groups[33–35]. In our study, we observed a diminution of the *Burkholderiales* and *Pasteurellales* order belonging to the *Proteobacteria* phylum, and a decrease in species of *Porphyromonas* belonging to the *Bacteroidetes* phylum in individuals who developed oral KS compared to control groups without oral KS. In parallel, there were increases in OTUs belonging to the *Firmicutes* phylum such as *Satelles* species, *Dialister* order and *Bacillaceae* family in the O-KS group (**Fig 4 and Fig 5**). Hence, the bacterial signature of oral KS is different from that of OSCC despite both types of cancer having an increase in OTUs of *Firmicutes* phylum and a decrease in OTUs of *Proteobacteria* phylum. However, the diminution of OTUs belonging to the *Bacteroidetes* phylum seems to be specific to individuals who developed oral KS and has not been described in other types of oral cancer. Moreover, as observed in the O-KS group, an increase of *Firmicutes* and decreases of *Bacteroidetes* and *Proteobacteria* phylotypes have previously been reported in other types of malignancies such as colorectal cancer (CRC)[36].

Various factors such as diet, oral hygiene, tobacco and alcohol consumption, stress, hormonal imbalance, diabetes, and gingival inflammation can perturb the oral bacterial community[37]. Studies have demonstrated that viral infections such as HIV, CMV, EBV and HSV-1 can also impact the composition of oral microbiome[38–40]. Other reports highlighted the negative effect of HIV load on microbiome. For examples, there were increases of *Porphyromonas* sp. and *Corynebacterium* order in HIV-infected individuals compared to HIV-negative individuals[41, 42].

We observed significant differences of some microbiota between individuals with neither oral KS nor detectable oral cell-associated KSHV DNA (No KSHV) and those with oral KS (O-KS) or detectable oral cell-associated KSHV DNA (O-KSHV) (**Fig 4 and Fig 5**). These results suggested that these differences were independent of KS status despite 5 of 9 individuals in the O-KS group had detectable oral cell-associated KSHV DNA (Table 1). Particularly, there was an increase in *Bacillales* OTU of the *Firmicutes* phylum in individuals with oral cell-associated KSHV DNA in the O-KSHV and O-KS groups. *Firmicutes* lineage has been associated with inflammation and cancer development in other reports. Indeed, *Clostridia* from *Firmicutes* lineage promoted carcinogenesis by inducing pro-inflammatory Th1 and Th17 immune responses in mice[43]. Moreover, anti-inflammatory responses can be induced by the generation of regulatory T cells through the production of short-chain fatty acid (SCFA) by bacteria belonging to the *clostridia* cluster[44–46]. Also, and perhaps even more important, SCFA, such as sodium butyrate and valproic acid, that act as histone deacetylase (HDAC) inhibitors, can reactivate KSHV[21, 22], and therefore increased butyrate production, might promote KS tumorigenesis through lytic activation of KSHV[47]. Interestingly, HIV-infected individuals with severe periodontal disease display a higher level of SCFA in the saliva compared to healthy individuals[48]. *Firmicutes* abundance has also been linked to TNF-α serum concentration in young obese patients[49]. However, since our study was cross-sectional, it is also possible that changes in the oral microbiome were a consequence of detectable oral cell-associated KSHV DNA or oral KS development, which is closely linked to the immune status of the subjects.

This study of the oral bacterial signature in AIDS-KS highlights for the first time the link between the oral microbiota and oral KS. A broader evaluation of the microbiome in the context of the development of visceral and systemic KS as well as resolution with immune reconstitution following antiretroviral therapy is warranted and may lead to novel biomarkers or probiotic approaches to treating KS.

## Materials and methods

### Study design and individual selection

We conducted a cross sectional analysis of the oral microbiome in twenty-nine individuals with pathology-confirmed KS who were serologically positive for KSHV and HIV, seen from December 2004 to February 2015 as part of clinical research protocols in the HIV and AIDS Malignancy Branch of the National Cancer Institute (NCI), which allowed the evaluation of oral cell-associated KSHV DNA. Individuals were selected from cohorts of AIDS-KS patients who were participating in clinical studies. Participants with at least 3 oral cell-associated KSHV DNA measurements were included. KS diagnosis was initially made by the attending physicians and confirmed by pathological examination by NCI pathologists with a biopsy by immunohistochemistry and sometimes by PCR for the presence of KSHV DNA. The KS stage of all patients was determined using the AIDS Clinical Trials Group Network (ACTG) criteria[50]. Presence or absence of macroscopically visible KS lesions in the oral mucosa was obtained from records of research physicians who performed physical examinations and was supported by photography. Individuals were classified into three groups based oral cell-associated KSHV DNA patterns and clinical documentation: oral KS with any oral cell-associated KSHV DNA status (O-KS, n = 9); no oral KS but with detectable oral cell-associated KSHV DNA (O-KSHV, n = 10); and with neither oral KS nor detectable oral cell-associated KSHV DNA over 3 or more time points (No KSHV, n = 10). Intermittent oral cell-associated KSHV DNA was defined based on the detection of KSHV DNA in oral cells in longitudinal follow-ups in at least 3 visits. Oral microbiota, oral cell-associated KSHV DNA, KSHV blood DNA detected in PBMC, and HIV load were examined at the time of sampling while CD4+ T cell count, CD8+ T cell count, the type of HIV therapy and duration, and years since HIV diagnosis were obtained from medical records. All participants provided written informed consent.

### Sampling and DNA extraction

Approximately 5 ml of Scope mouthwash was used to collect oral fluids and cellular materials. Samples were centrifuged at 8,000 g for 5 min to pellet cellular materials. The supernatant was transferred to another tube without disturbing the pellet. Pellets were stored at -80 ^o^C prior to extraction. PBMC materials were matched with oral cells (same draw date) in every incidence except one intermittent shedder who had PBMC draw next day and for two never shedders who had PBMC materials within a month of the collection of oral cells. PBMC were isolated from blood collected in acid citrate dextrose (ACD) tubes by Ficoll (GE Healthcare) centrifugation with Leucosep tubes (Greiner bio-one). Red blood cells were lysed with ammonium-chloride-potassium (ACK) buffer following manufacturer’s protocol (Thermo Scientific). The purified PBMC were counted using a Nexcellom Cellometer Vision instrument. Pellets of approximately 1–2 million PBMC were obtained for each individual and stored at -80°C prior to DNA extraction.

Genomic DNA was extracted from oral cells and PBMC pellets using QIAamp Blood Mini Kits according to the manufacturer’s instructions (Qiagen). The quality and quantity of the DNA extracted were measured using a UV spectrophotometer at 260 and 280 nm (Nanodrop 2000). All DNA samples were stored at -80 ^o^C.

### Measurements of KSHV DNA and antibodies

KSHV DNA in oral cell pellet and PBMC collected at the time of oral sampling were measured as previously described[51]. Briefly, KSHV DNA was measured in a real-time PCR assay based on the ORF-K6 gene. Triplicate assays were performed, and the averages of the triplicate values were used to determine the viral copies. The cell-associated KSHV DNA values were converted to copies per million cells using a cell quantitation assay based on the ERV-3 gene[52] with an assay sensitivity of 3 copies/10^6^ cells. The assays are Clinical Laboratory Improvement Amendments (CLIA) certified and conducted using stringent procedures to prevent contamination.

KSHV serological status was determined using recombinant protein ELISA for ORF73 and K8.1. Participants were considered KSHV-seropositive if they had antibodies to either antigen.

### Next-generation sequencing

The first PCR amplification of the bacterial 16S rRNA gene hypervariable V1-V2 region was performed with DNA isolated from oral cell pellet using the forward primer 5’-TCGTCGGCAGCGTCAGATGTGTATAAGAGACAG-3’ and the reverse primer 5’-GTCTCGTGGGCTCGGAGATGTGTATAAGAGACAG-3’, both of which contain overhang adapters. The PCR reaction was carried out by 2 min initial denaturation at 95 ^o^C, 25 cycles of 30 sec denaturation at 95 ^o^C, 30 sec elongation at 72 ^o^C, and a 5 min one final extension at 72 ^o^C. Illumina sequencing adapters and indexes were attached using Nextera XT Index kit (Cat. FC-131-1001, Illumina). The amplicon library was built and sequenced with the Illumina MiSeq V3 600 Cycle Kit according to the manufacturer’s instructions (Illumina) using the universal 16S primers 27F (5'-AGAGTTTGATCMTGGCTCAG-3’) and 355R (5'-GCTGCCTCCCGTAGGAGT-3’)[53].

### Bioinformatics and statistical analysis

Raw pyrosequencing results from the hypervariable region V1-V2 of the 16S rRNA gene were filtered and unique reads were assigned to the corresponding samples after mapping with barcode and primer sequences. FastQC was used to evaluate the sample reads for sequencing quality[54]. Sequences were analyzed using quantitative insights into microbial ecology (QIIME) software[55]. Sequences were assigned to operational taxonomic units (OTUs) at 97% similarity at different taxonomic levels (from phylum to species) to the Human Oral Microbiome Database with a 97% cutoff value. Bacterial diversity was determined by performing a sampling-based OTU analysis and was displayed as rarefaction curves (Shannon index curves). Bacterial richness and diversity across samples were analyzed using the following α indexes: Shannon, Chao, Ace and Observed species. Comparison of OTUs abundance across groups was performed using Student’s t-test with the GraphPad Prism software[56].

For all statistic tests, P-value ≤0.05 (*), ≤0.005 (**) or ≤0.0005 (***) were considered significant.

### Ethics statement

All patients were enrolled on National Institutes of Health Clinical Center Protocol 01-C-0038 (registered in clinicaltrials.gov as NCT00006518), which was approved by the National Cancer Institute Institutional Review Board, and the samples were obtained and studied as part of this protocol. All individuals gave written informed consent to clinical examination, sample acquisition, and testing on clinical samples. However, all the data were analyzed anonymously.

## Supporting information

S1 Fig Taxonomic compositions of bacterial communities at phylum and species levels for each individual HIV/KSHV-coinfected subject. (A) Relative OTU abundances at phylum level clustered by each individual subject. (B) Relative OTU abundances of the 30 most abundant species clustered by each individual subject.
(TIF)
